# Study on the Thermal Stabilizing Process of Layered Double Hydroxides in PVC Resin

**DOI:** 10.3390/molecules28237792

**Published:** 2023-11-27

**Authors:** Zhi Rao, Kaitao Li, Pingli Liu, Yanjun Lin, Xiang Lyu

**Affiliations:** 1 Department of Applied Engineering, Gandong College, Fuzhou 344000, China; 2 State Key Laboratory of Chemical Resource Engineering, Beijing University of Chemical Technology, Beijing 100029, China; 3 Electrification and Energy Infrastructures Division, Oak Ridge National Laboratory, Oak Ridge, TN 37831, USA

**Keywords:** LDHs, thermal stabilizing process, PVC, thermal stabilizer

## Abstract

Poly(vinyl chloride) (PVC) is widely used in various fields and requires the use of thermal stabilizers to enhance its thermal stability during processing because of its poor thermal stability. Layered double hydroxides (LDHs) are widely considered to be one kind of highly efficient and environmentally friendly PVC thermal stabilizer. To investigate the thermal stabilizing process of layered double hydroxides (LDHs) in PVC resin, PVC and MgAl-LDHs powders with different interlayer anions (CO_3_^2−^, Cl^−^, and NO_3_^−^) were physically mixed and aged at 180 °C. The structure of LDHs at different aging times was studied using XRD, SEM, and FT-IR. The results show that the thermal stabilizing process of LDHs on PVC mainly has three stages. In the first stage, the layers of LDHs undergo a reaction with HCl, which is released during the thermal decomposition of PVC. Subsequently, the ion exchange process occurs between Cl^−^ and interlayer CO_3_^2−^, resulting in the formation of MgAl-Cl-LDHs. Finally, the layers of MgAl-Cl-LDHs react with HCl slowly. During the thermal stabilizing process of MgAl-Cl-LDHs, the peak intensity of XRD reduces slightly, and no new XRD peak emerges. It indicates that only the first step happens for MgAl-Cl-LDHs. The TG-DTA analysis of LDHs indicates that the interaction of LDHs with different interlayer anions has the following order: NO_3_^−^ < CO_3_^2−^ < Cl^−^, according to the early coloring in the thermal aging test of PVC composites. The results of the thermal aging tests suggest that LDHs with a weak interaction between interlayer anions and layers can enhance the early stability of PVC significantly. Furthermore, the thermal aging test demonstrates that LDHs with high HCl absorption capacities exhibit superior long-term stabilizing effects on PVC resin. This finding provides a valuable hint for designing an LDHs/PVC resin with a novel structure and excellent thermal stability.

## 1. Introduction

Polyvinyl chloride (PVC), which was successfully synthesized in the laboratory in 1872, is considered one of the earliest thermoplastic polymers. PVC has several desirable characteristics, including refractory combustion, good mechanical properties, affordability, abundant raw materials, and well-established synthesis technology. As a result, it is widely used in various industries such as construction, telecommunications, transportation, electronics, the chemical industry, packaging, and others. However, PVC has a strong tendency to degrade when exposed to heat or radiation (such as UV light) during melt-processing and practical applications [1,2]. HCl originated from the thermal dehydrochlorination of PVC chains and is believed to sustain this auto-catalytic process, resulting in unacceptable discoloration and a drastic change in the physical and mechanical properties of the resin [3,4]. Due to the poor thermal stability of PVC, there is only a small difference between its softening temperature and decomposition temperature, which poses significant challenges during processing. Therefore, it is necessary to add an appropriate number of stabilizers that can irreversibly bond HCl and capture unstable Cl during the processing stage to enhance its thermal stability [5,6]. At present, the main classes of thermal stabilizers used for PVC are lead salts, metal soaps, and organic compounds [7]. Nevertheless, they have obvious disadvantages in terms of their toxicity, environmental pollution, efficiency, and cost.

In 1981, the Kyowa Chemical Industries of Japan reported a patent that first indicated a significant improvement in the performance of PVC composites by two-dimensional (2D)-layered double hydroxides (LDHs) [8]. Since then, LDHs have attracted extensive attention and have been used as a popular commercial thermal stabilizer for PVC resin in China, Japan, and Europe [9,10]. This has resulted in excellent performance, low costs, and non-toxic PVC fillers [11,12]. The structure of LDHs is similar to brucite (Mg(OH)_2_). The unit layer is formed by the shared edges of the MgO_6_ octahedron. Aluminum can replace magnesium within a certain range, resulting in positively charged laminae. The exchangeable CO_3_^2−^ between the layers balances the positive charge, making the overall structure of LDHs electrically neutral. The interlayer anions of LDHs are exchangeable due to the hydrogen bond connection between the lamella and interlayer anions. Additionally, LDHs contain interlayer water molecules that can be removed without damaging the layer’s structure. The general formula of LDH is [M^2+^_1−x_M^3+^_x_(OH)_2_](A^n−^)_x/n_·mH_2_O, where M^2+^ and M^3+^ represent the divalent and trivalent metallic cations, respectively; A^n−^ is an exchangeable n-valent anion, and x is equal to the value of the molar ratio M^3+^/(M^2+^ + M^3+^); and m is the number of interlayer water molecules [13,14]. Because of this structure, LDHs can effectively absorb the HCl formed during the dehydrochlorination of PVC and hinder the autocatalytic thermal decomposition reactions [15,16]. In addition, LDH materials have received considerable attention due to their potential applications in nanocomposite hydrogel, rheology, UV-light shielding, oil-enhanced recovery, catalysts, electrodes, and anionic exchangers [17,18,19,20,21,22,23,24]. Over the past few decades, the effects of different Mg/Al ratios, intralayer cations, the substitution of interlayer anions, and the surface modification of LDHs on the thermal stability property of PVC have been investigated in detail in the literature [25,26,27,28]. However, few experimental works have been performed to study the thermal stabilizing mechanism of LDHs for PVC.

It was first speculated that the stabilizing process involves the following two steps: the interlayer anions initially react with HCl, then the layers react with HCl to form metal chlorides with the loss of the layered structure [29]. Later research, including our previous work, is based on this unproved mechanism [30], and no further process and mechanism have been studied, partly because it is hard to study the structural change in LDHs in PVC composite, which contains many necessary additives to be blended at more than 100 °C. Therefore, how to improve the stabilizing effect of LDHs on PVC, such as early coloring and long-term stability, remains unclear, which hinders the improvement of the thermal stabilizing effect of LDHs using abundant controllable properties of the composition and the structure of LDHs.

In this paper, LDH samples were prepared using a method involving separate nucleation and aging steps (the SNAS method) developed in our laboratory. The complete crystal structure of MgAl-LDHs with different interlayer anions (CO_3_^2−^, Cl^−^, and NO_3_^−^) was successfully obtained. Then, the thermal stabilities of composites containing the PVC resin, LDHs, Ca(st)_2_, Zn(st)_2,_ and DOP were compared. To understand the thermal stability mechanism of LDHs on PVC, LDH powders with different interlayer anions were physically mixed with PVC powder without any other additives or heating, followed by thermal aging at 180 °C. The structural changes in LDHs at different times could be studied without the influence of other additives to evaluate the stabilizing process of LDHs on the PVC resin. The difference in the early and long-term stabilizing effect of LDHs was also studied by analyzing the reactivity of the OH group with HCl and the total HCl absorption capacities.

## 2. Results and Discussion

### 2.1. Structure of MgAl-LDHs

**Figure 1:**
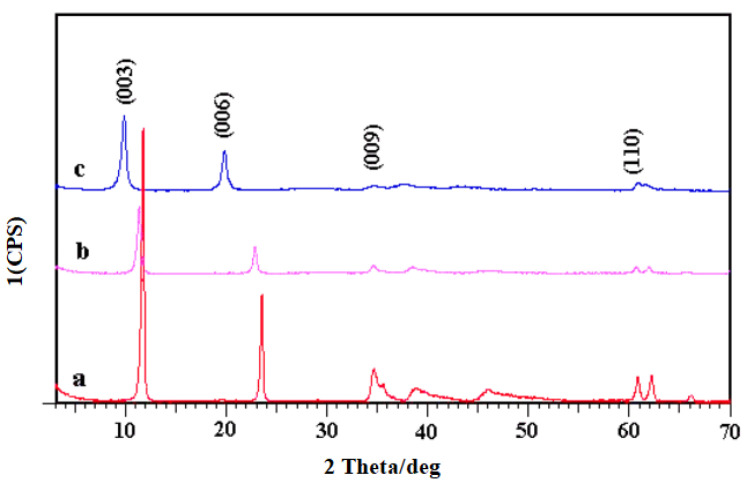
XRD patterns of (a) MgAl-CO_3_-LDHs, (b) MgAl-Cl-LDHs, and (c) MgAl-NO_3_-LDHs.

The XRD patterns of MgAl-LDHs with different interlayer anions (CO_3_^2−^, Cl^−^, and NO_3_^−^) are shown in Figure 1. As shown in Figure 1, all these patterns have the characteristic features of layered materials [31]. The LDH phases (JCPDS file no. 38-0487) were observed as the major phase in all samples [32]. The (003) crystal plane diffraction peaks of LDHs with interlayer anions of CO_3_^2−^, Cl^−^, and NO_3_^−^ appeared at 2θ = 11.7°, 11.3° and 9.8°. The peaks near 61° correspond to the (110) crystal plane. The strong and sharp diffraction peaks indicate that these MgAl-LDHs had relatively well-formed crystalline structures. No impurity peak was observed in these XRD patterns.

### 2.2. Thermal Analysis of MgAl-LDHs

**Figure 2:**
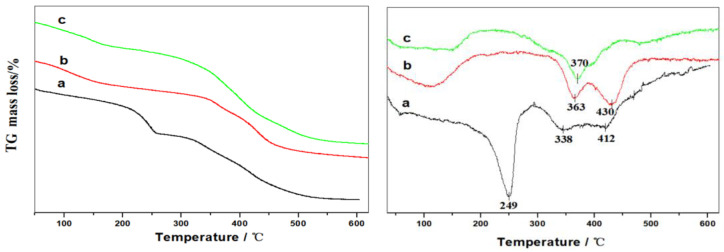
TG-DTA curves of (a) MgAl-CO_3_-LDHs, (b) MgAl-Cl-LDHs, and (c) MgAl-NO_3_-LDHs.

The TG-DTA curves of MgAl-LDHs with different interlayer anions (CO_3_^2−^, Cl^−^, and NO_3_^−^) are displayed in Figure 2. It is observed that the thermal decomposition behaviors of these LDHs are similar. The weight loss process of these LDHs consists of the following three stages: the removal of interlayer crystal water, the dehydroxylation of the layers, and the loss of interlayer anions [33]. The three DTA peaks of MgAl-CO_3_-LDHs occurred at 249 °C, 338 °C, and 412 °C, respectively. The boundary between the second and the third stage of MgAl-NO_3_-LDHs is not obvious, with DTA peaks occurring around 320 °C and 370 °C.

Based on the DTA curves, it can be observed that although these LDHs have the same layers, the third stage of removing interlayer anion occurred at different temperatures: approximately 412 °C, 430 °C, and 370 °C, respectively. A lower temperature indicated that the removal of interlayer anions was easier, suggesting weaker interactions between these anions and the layers. Additionally, the different interactions also impact the dehydroxylation of the layers, while the DTA peaks of the second stage shift to higher temperatures in the following order: NO_3_^−^ < CO_3_^2−^ < Cl^−^. The lower temperature of dehydroxylation implies that the removal of OH in the layers is easier, which indicates that these OH groups could exhibit a higher reactivity with HCl.

### 2.3. Thermal Stabilizing Process of MgAl-CO_3_-LDHs in PVC

**Figure 3:**
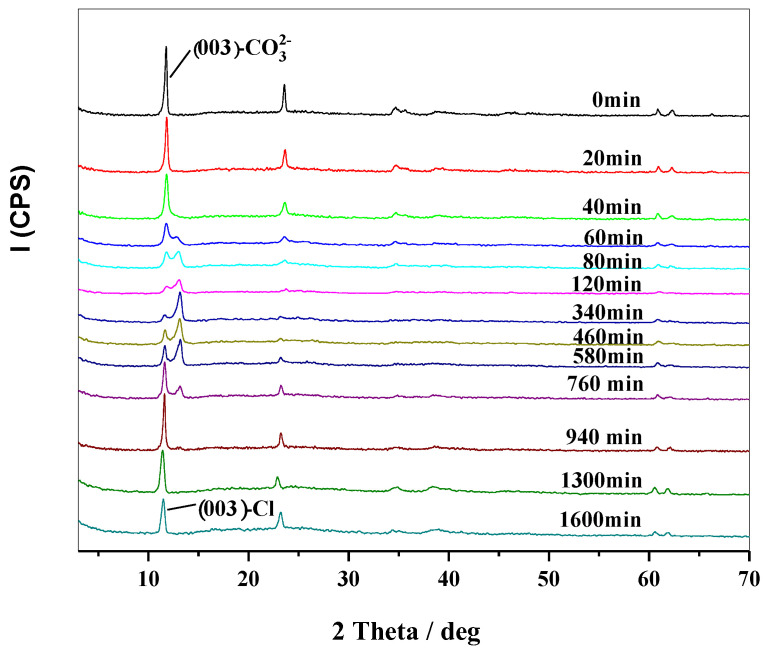
XRD patterns of PVC + MgAl-CO_3_-LDHs aged at 180 °C.

To investigate the effects of thermal aging on the crystal phase, a mixture of 2 g PVC powder and 1 g MgAl-CO_3_-LDH powder was mixed and placed in a thermal aging test box at 180 °C. At different time intervals, the mixture was taken out and subjected to XRD characterization. The peak intensity in XRD analysis indicates the content and structure of the crystal phase. It can be seen in Figure 3 that the peak intensities of MgAl-CO_3_-LDHs decreased gradually before 60 min, while the full width at half maximum (FWHM) increased slowly. This trend suggests that the LDH layers underwent a reaction with HCl released by PVC during the thermal aging process, resulting in a reduction in the particle size of LDHs. From 60 min to 340 min, the intensity of the (003) peak at about 11.7° (d = 0.75 nm) decreased gradually, while a new peak appeared at about 12.7° (d = 0.69 nm) with increasing peak intensity. This occurrence signifies the gradual removal of interlayer water and a subsequent decrease in the interlayer space.

From 340 min to 940 min, a new peak at about 11.6° (d = 0.76 nm) emerged and increased in height over time. Concurrently, the intensity of the peak at 12.7° decreased slowly. This could be attributed to the ion-exchange process of Cl^−^ with CO_3_^2−^, resulting in the formation of MgAl-Cl-LDHs with a (003) peak at about 11.6° [34]. The peak at 12.7° disappeared after 940 min, indicating that the interlayer of CO_3_^2−^ was exchanged via Cl^−^ completely. Then, the peak intensity of the formed MgAl-Cl-LDHs decreased slowly with the aging time, while the FWHM increased slightly. This suggests that LDHs with the interlayer Cl^−^ found it difficult to react with the HCl given off by PVC.

**Figure 4:**
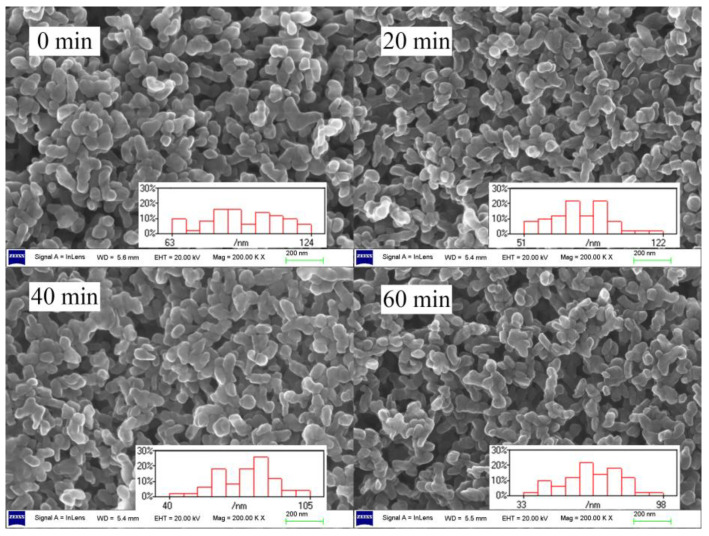
SEM of LDHs at early aging time.

**Figure 5:**
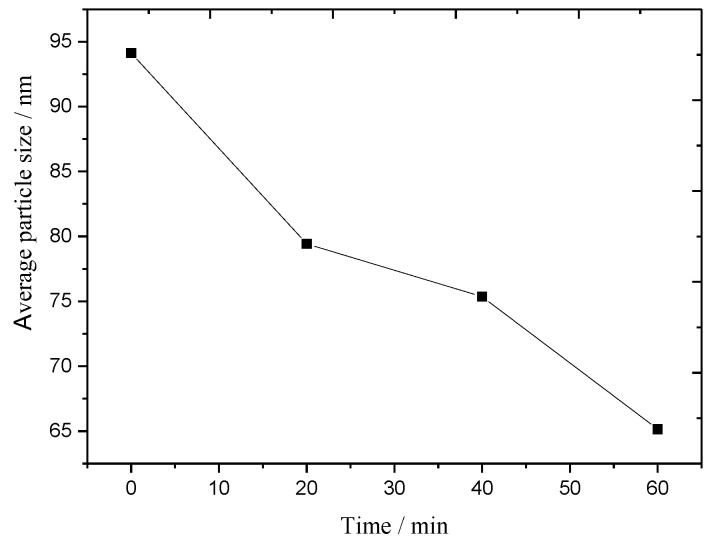
Average particle size of LDHs at early aging time.

In order to observe the change in particle size of the above LDH samples aged from 0 min to 60 min, a small amount of the mixture was dissolved in tetrahydrofuran to remove the PVC resin every time after XRD characterization. The residual LDHs were tested using SEM. The SEM of LDHs at early aging time is shown in Figure 4. It can be seen that the structure of LDHs remained similar before and after thermal aging, with small and uniform particle sizes. The primary particle size distribution ranged from 30 nm to 120 nm. We measured LDH particles from each image with Nano Measurer 1.2 software and obtained a corresponding particle size histogram. After calculation, the average particle size of residual LDHs under different aging times is presented in Figure 5. The figure clearly shows that, as the aging time increases, the average particle size of LDHs significantly decreases from 94 nm to 65 nm, which is consistent with the XRD results.

**Figure 6:**
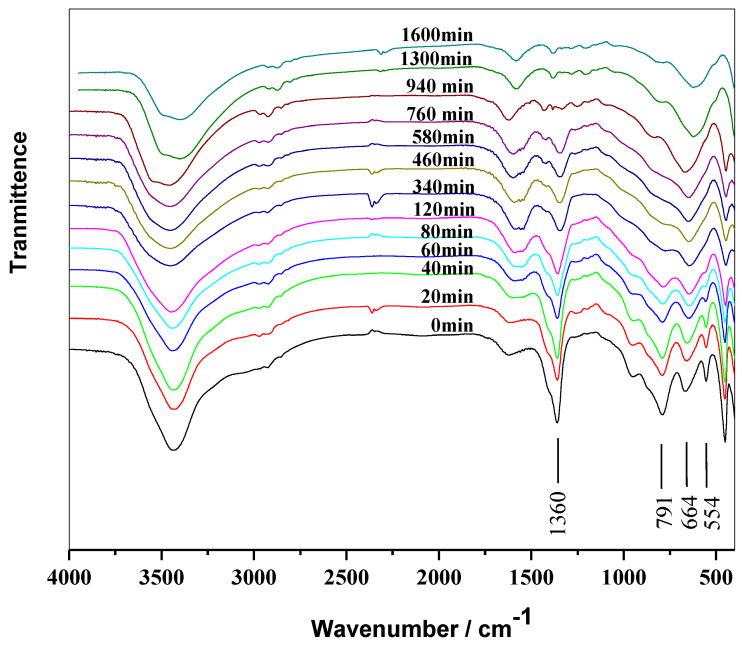
FT-IR patterns of PVC + MgAl-CO_3_-LDHs aged at 180 °C.

After being dissolved in tetrahydrofuran to remove the PVC resin, the residual LDHs were dried and measured via FT-IR. As shown in Figure 6, the stretching vibration peaks of Al-O and Mg-O at 947, 791, 664, and 554 cm^−1^ decreased with the aging time and tended to disappear after 340 min. This could be attributed to the reaction between the layers with the surface OH group and the HCl generated during the thermal decomposition of PVC, leading to gradual damage to the layer. These findings are consistent with the results obtained from XRD and SEM analyses. The gradual weakening and eventual disappearance of the antisymmetric ν_3_ vibration of CO_3_^2−^ at 1361 cm^−1^ after 940 min could be attributed to the complete exchange of the interlayer CO_3_^2−^ with Cl^−^ to form MgAl-Cl-LDHs [35]. These results are also consistent with the XRD patterns in Figure 3. After 940 min, there was a minimal change in the peaks, indicating that LDHs with the interlayer Cl^−^ are difficult to react with HCl.

Therefore, it can be concluded that the thermal stabilizing process of MgAl-CO_3_-LDHs on PVC can mainly be divided into three stages. In the first stage, LDH layers react with the HCl released during PVC thermal decomposition, resulting in a decrease in the particle size of LDHs. In the second stage, Cl^−^ exchanges with the interlayer CO_3_^2−^, leading to the formation of MgAl-Cl-LDHs. In the third stage, MgAl-Cl-LDH layers react with HCl slowly.

### 2.4. Thermal Stabilizing Process of MgAl-NO_3_-LDHs in PVC

**Figure 7:**
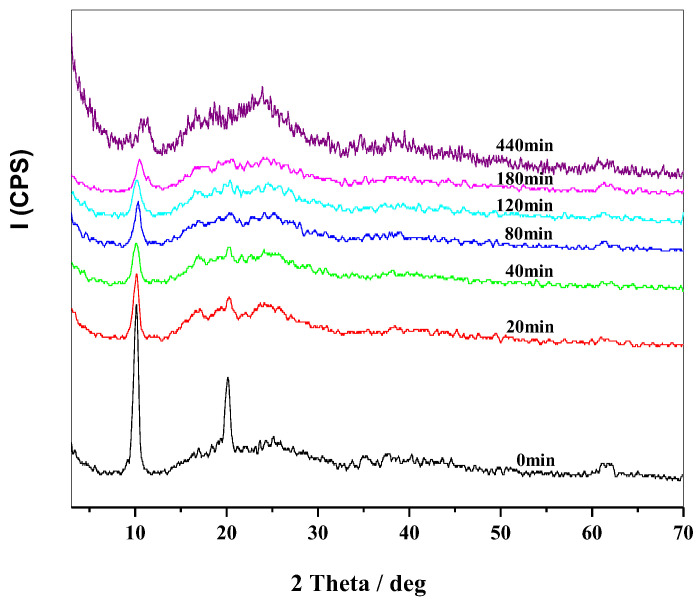
XRD patterns of PVC + MgAl-NO_3_-LDHs aged at 180 °C.

A mixture of 2 g PVC powder and 1 g MgAl-NO_3_-LDHs powder was prepared and placed in a thermal aging test box at 180 °C. The mixture was then taken out at different time intervals and subjected to XRD analysis. The peak intensity in XRD analysis indicates the content and structure of the crystal phase. As shown in Figure 7, the peak intensity of MgAl-NO_3_-LDHs decreases gradually, and the FWHM increases with the aging time. This result is similar to that of MgAl-CO_3_-LDHs, indicating that LDH layers react with the HCl released by PVC, resulting in a decrease in the particle size of LDHs. Additionally, the (003) peak moves slightly to a higher angle after 120 min from 9.8° to about 10.9°. This is the result of the removal of interlayer water under 180 °C, which decreases the layer’s space. Compared with Figure 3, it is clear that the peak intensity of MgAl-NO_3_-LDHs decreases at a faster rate with the aging time than MgAl-CO_3_-LDHs. This indicates that the reaction rate between MgAl-NO_3_-LDHs and HCl is faster than that of MgAl-CO_3_-LDHs. Moreover, it can also be noted from Figure 7 that the (003) peak remains lower than 11.3°, indicating that Cl^−^ released from PVC can not be exchanged with NO_3_^−^. This is due to the stronger acidic nature of nitric acid compared to hydrochloric acid, preventing the exchange of Cl^−^ with NO_3_^−^ under these conditions.

### 2.5. Thermal Stabilizing Process of MgAl-Cl-LDHs in PVC

**Figure 8:**
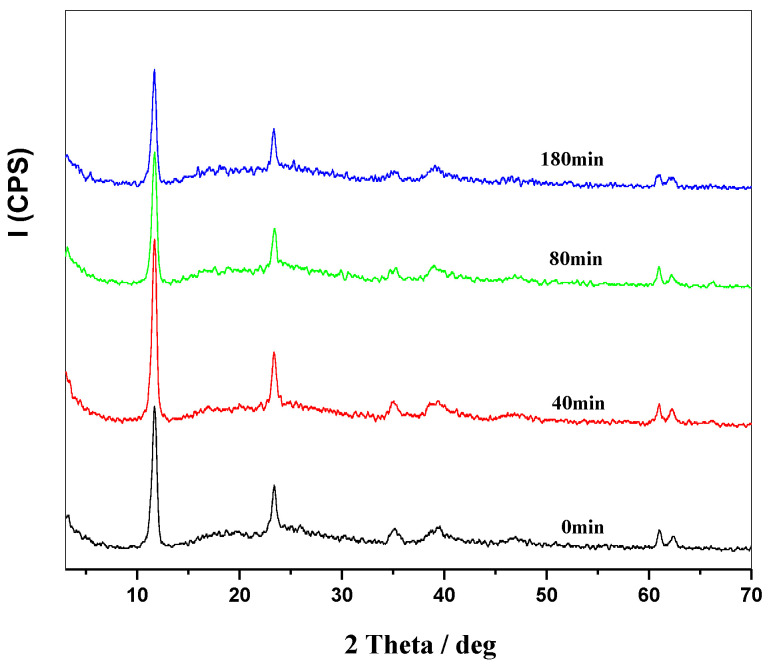
XRD patterns of PVC + MgAl-Cl-LDHs at 180 °C.

A mixture of 2 g of PVC powder and 1 g of MgAl-Cl-LDHs powder was prepared and placed in a thermal aging test box at 180 °C. The mixture was then taken out at different time intervals and subjected to XRD analysis. The results are shown in Figure 8. The peak intensity in XRD analysis indicates the content and structure of the crystal phase. A higher peak intensity suggests a larger number or a better quality of the crystals. During the thermal stabilization process of MgAl-Cl-LDH in PVC, the peak intensity exhibited a slight decrease over time, indicating a weak reaction between LDH laminates and HCl released by the thermal decomposition of PVC. Furthermore, Figure 8 illustrates that no new characteristic diffraction peak emerged over an extended period, indicating the absence of the anion exchange reaction between the layers.

### 2.6. Thermal Stabilizing Effect of Different MgAl-LDHs in PVC

**Figure 9:**
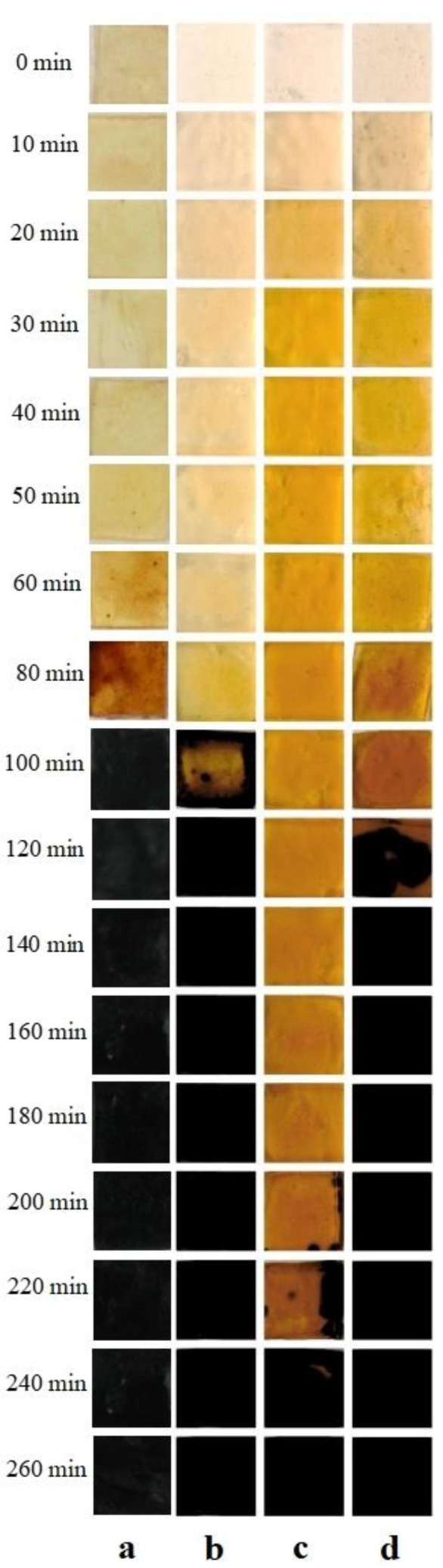
Thermal stabilizing effect of (**a**) Ca(st)_2_ + Zn(st)_2_, (**b**) Ca(st)_2_ + Zn(st)_2_ + MgAl-NO_3_-LDHs, (**c**) Ca(st)_2_ + Zn(st)_2_ +MgAl-CO_3_-LDHs, and (**d**) Ca(st)_2_ + Zn(st)_2_ + MgAl-Cl-LDHs on PVC.

The thermal stabilities of composites containing the PVC resin, Ca(st)_2_, Zn(st)_2,_ and DOP with different LDHs are compared in Figure 9. As shown in Figure 9, the PVC sample, containing only calcium and a zinc heat stabilizer, turned black in approximately 60 min. The thermal stability was found to be inferior to that of PVC-containing composite thermal stabilizers with LDHs. This suggests that LDHs and calcium and zinc heat stabilizers exhibit a positive synergistic effect on stability. It can also be seen that LDHs with different interlayer anions have different stabilizing effects on PVC. MgAl-CO_3_-LDHs possess the best long-term stabilizing effect on PVC, while the PVC sample blackens at about 180 min. The stability time of PVC containing MgAl-NO_3_-LDHs and MgAl-Cl-LDHs are 80 min and 100 min, which are much lower than that of MgAl-CO_3_-LDHs. This is because both layers and interlayer ions of MgAl-CO_3_-LDHs have the ability to react with and absorb Cl^−^ released from PVC, and only the layers of MgAl-NO_3_-LDHs and MgAl-Cl-LDHs can react with and absorb Cl^−^. It can also be seen from Figure 9 that the color of PVC strips containing MgAl-NO_3_-LDHs is clearly lighter than the others, which means better early coloring, indicating that MgAl-NO_3_-LDHs have a better thermal stabilizing effect on the PVC resin in early aging time.

**Figure 10:**
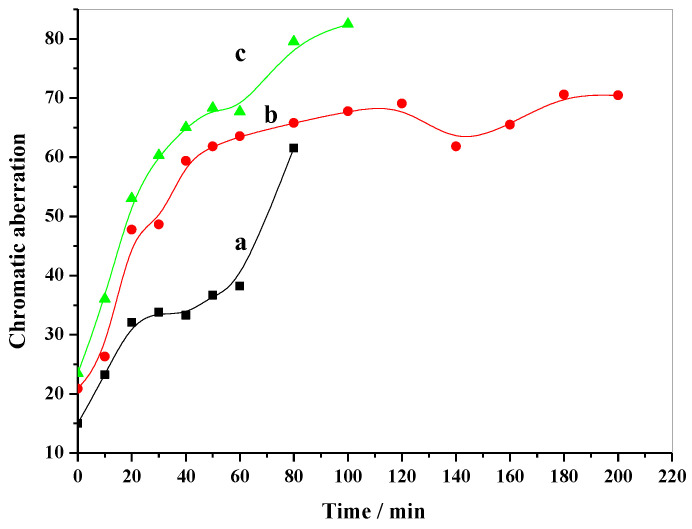
Chromatic aberration curves of the thermal stabilizing effect of (**a**) MgAl-CO_3_-LDHs, (**b**) MgAl-Cl-LDHs, and (**c**) MgAl-NO_3_-LDHs on PVC.

The color tests of these PVC strips were carried out using a colorimeter, and the results for the chromatic aberration relative to a standard MgO white plate are shown in Figure 10. It can be observed that PVC strips containing MgAl-NO_3_-LDHs exhibited better chromatic aberration than the others before 80 min, while PVC strips containing MgAl-Cl-LDHs showed the highest chromatic aberration before 100 min. This result is consistent with Figure 9. Additionally, PVC strips containing MgAl-CO_3_-LDHs had a slightly lower chromatic aberration compared to MgAl-Cl-LDHs, which means only a slightly better early coloring and stabilizing effect on PVC. As analyzed using the TG-DTA curves of LDHs (Figure 2), NO_3_^−^ experienced a weaker interaction with the layers, resulting in the greater reactivity of the layers of MgAl-NO_3_-LDHs in absorbing HCl during the early aging stage, leading to the improved early coloring of the PVC composite. On the other hand, Cl^−^ has a stronger interaction with the layers, resulting in the poorer early coloring of PVC containing MgAl-Cl-LDHs. These results also indicate that the absorption of HCl by the layers is important in early aging time, while the reaction between LDH layers and HCl mainly happens in the first stage.

The theory of HCl absorption capacities of different MgAl-LDHs is listed in Table 1. Among these three LDHs, MgAl-CO_3_-LDHs exhibit the best total HCl adsorption capacity, resulting in the longest stabilizing time on PVC. However, in the early aging stage, its stabilizing effect is worse than MgAl-NO_3_-LDHs. This is because the layers of MgAl-NO_3_-LDHs have higher reactivity in absorbing HCl, as discussed when using TG-DTA and chromatic aberration. The HCl adsorption capacity of MgAl-NO_3_-LDHs is less than the other LDHs; therefore, its long-term stabilizing time is shorter.

## 3. Materials and Methods

### 3.1. Materials

A.R. grade of NaOH, Na_2_CO_3_, Mg(NO_3_)_2_·6H_2_O, Al(NO_3_)_3_·9H_2_O, MgCl_2_·6H_2_O, AlCl_3_·6H_2_O were obtained from the Beijing Chemical Co., Ltd. (Beijing, China) CO_2_-free deionized water with a conductivity less than 10^−6^ S·cm^−1^ was used in synthesis and washing steps. Dioctylphthalate (DOP), calcium stearate (Ca(St)_2_), and zinc stearate (Zn(St)_2_) were all of the C.P. grade. The PVC resin used was SG5 with a limited viscosity number of 107–118 mL·g^−1^ and an average polymerization degree of 1000–1100.

### 3.2. Preparation of LDHs

LDH samples were prepared using a method involving separate nucleation and aging steps (the SNAS method) developed in our laboratory [36,37]. A solution of Mg(NO_3_)_2_·6H_2_O and Al(NO_3_)_3_·9H_2_O with Mg/Al molar ratios of 2.0 was prepared with the concentration of Mg^2+^ fixed at 0.8 mol/L. A mixed base solution containing NaOH and Na_2_CO_3_ was prepared with n(CO_3_^2−^)/n(M^3+^) = 2 and n(NaOH)/[n(M^2+^) + n(M^3+^)] = 1.6. These two solutions were mixed in a colloid mill to obtain a slurry containing LDHs, followed by aging for 6 h at reflux temperature. Then, the precipitate was washed and dried at 70 °C for 24 h to obtain MgAl-CO_3_-LDHs powders. MgAl-NO_3_-LDHs were prepared using the same method without Na_2_CO_3_. MgCl_2_·6H_2_O, AlCl_3_·6H_2_O, and NaOH were used to prepare MgAl-Cl-LDHs.

### 3.3. Characterization of Samples

Powder X-ray diffraction patterns were recorded on a Shimadzu XRD-6000 diffractometer (Shimadzu, Kyoto, Japan), using Cu Kα radiation (λ = 0.15406 nm) at 40 kV and 30 mA, with a scanning rate of 10°·min^−1^, in a 2θ range from 3° to 70°. FT-IR spectra were obtained on a Bruker Vector 22 Fourier transfer infrared spectrophotometer (Bruker, Karlsruhe, Germany) using the KBr disk method with a sample/KBr ratio of 1:100 by mass. Thermogravimetry and differential scanning calorimetry (TG-DTA) curves were recorded on a Beifen PCT-IA instrument (Beifen, Beijing, China) at the temperature range of 25–700 °C with the heating rate at 10 °C·min^−1^ in the air. The morphology of the samples was observed using a supra55 scanning electron microscope (SEM) (Carl Zeiss, Oberkochen, Germany), and all the samples were treated with gold spraying.

### 3.4. Thermal Stability Testing of PVC Strips

Mixtures containing 100 g of PVC powder, 50 g of DOP, 2.3 g of Ca(st)_2_, 0.7 g of Zn(st)_2,_ and 2 g of different LDHs were blended in a heated double-roller mixer for 10 min at 140 °C. The resulting mixtures were compression-molded at 100 °C to a film with a thickness of 1mm and cut into 1 cm × 1 cm strips. These strips were placed in a thermal aging test box at 180 ± 1 °C and subjected to static thermal aging according to the ISO standard [38]. Strips were removed at different times and subjected to chromatic aberration examination by a colorimeter.

## 4. Conclusions

The thermal stabilizing process of LDHs with different interlayer anions (CO_3_^2−^, Cl^−^, and NO_3_^−^) in PVC resin was investigated extensively in this work. The thermal stabilizing process of LDHs on PVC had mainly three stages based on these results. Firstly, LDH layers reacted with the HCl released from PVC. Secondly, the Cl^−^ exchange with interlayer CO_3_^2−^ led to the formation of MgAl-Cl-LDHs. Thirdly, MgAl-Cl-LDH layers reacted with HCl slowly. In case Cl^−^ could not exchange with the original ions, only the first step took place. LDHs with weak interaction between interlayer anions and layers could enhance the early stability of PVC markedly, while the LDHs with higher HCl absorption capacities had better long-term stabilizing effects on PVC. In addition, during the early stage of PVC thermal aging, the LDH thermal stabilizer exhibited a decrease in the grain size on all sides. This decrease can be attributed to the erosion of LDH laminates using HCl, which is produced during PVC degradation and reacts with LDHs. These findings are helpful in clarifying the stabilizing process and mechanism of LDHs on PVC and in designing effective LDH thermal stabilizers with novel compositions and structures.

## Figures and Tables

**Table 1:** Theoretical HCl absorption capacities of LDHs with different interlayer anions.

Theoretical HCl Absorption Capacities (mmol HCl/g LDH)	NO_3_^−^	CO_3_^2−^	Cl^−^
Layers	21.77	24.63	24.09
Interlayer	/	4.10	/
Total	21.77	28.7	24.09

## Data Availability

Data are contained within the article.
